# Translesion DNA synthesis on pyrimidine dimers by plant organellar DNA polymerases is metal-dependent

**DOI:** 10.1093/nar/gkag707

**Published:** 2026-07-14

**Authors:** Noe Baruch-Torres, Joon Park, Eduardo Castro-Torres, Shigenori Iwai, Y Whitney Yin, Luis G Brieba

**Affiliations:** Unidad de Genómica Avanzada, Centro de Investigación y de Estudios Avanzados del IPN, Apartado Postal 629, Irapuato, Guanajuato, CP 36821, México; Department of Biochemistry and Molecular Biology, University of Texas Medical Branch, Galveston, TX, United States; Sealy Center for Structural Biology and Molecular Biophysics, Galveston, TX, United States; Department of Biochemistry and Molecular Biology, University of Texas Medical Branch, Galveston, TX, United States; Sealy Center for Structural Biology and Molecular Biophysics, Galveston, TX, United States; Department of Biochemistry and Molecular Biology, University of Texas Medical Branch, Galveston, TX, United States; Division of Chemistry, Graduate School of Engineering Science, Osaka University, Toyonaka, Japan; Department of Biochemistry and Molecular Biology, University of Texas Medical Branch, Galveston, TX, United States; Sealy Center for Structural Biology and Molecular Biophysics, Galveston, TX, United States; Unidad de Genómica Avanzada, Centro de Investigación y de Estudios Avanzados del IPN, Apartado Postal 629, Irapuato, Guanajuato, CP 36821, México

## Abstract

Ultraviolet (UV) radiation generates DNA lesions, primarily cyclobutane pyrimidine dimers (CPDs) and 6–4 photoproducts ([6–4] PPs), that can block DNA replication. Although nuclear UV-induced lesions are repaired or bypassed by specialized pathways, how plant organellar DNA polymerases replicate UV-damaged templates remains unclear. Here, we show that the two *Arabidopsis thaliana* organellar DNA replicases, AtPolIs, efficiently synthesize across CPDs with 80%–90% bypass efficiency, exceeding that reported for individual specialized translesion synthesis (TLS) polymerases. Furthermore, although [6–4] PPs impose a major barrier to most TLS polymerases, wild-type AtPolIs exhibit measurable lesion-bypass activity (∼10%), and reduction of exonuclease activity enhances bypass by ~8-fold, reaching levels comparable to synthesis on undamaged templates. We further demonstrate that TLS across UV photoproducts depends on three unique amino acid insertions within the polymerase domain, as disruption of these insertions severely compromises lesion bypass. These findings reveal that AtPolIs are replicative polymerases with an intrinsic and unusually robust capacity for UV-lesion bypass, suggesting a specialized adaptation that helps maintain plant organellar genome stability under UV stress.

## Graphical Abstract

**Graphical Abstract:**
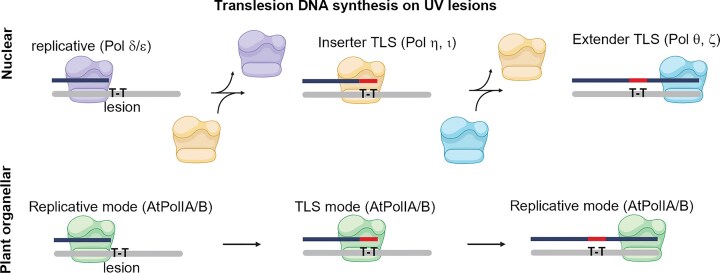

## Introduction

Ultraviolet-B (UV-B) radiation (280–315 nm), a ubiquitous DNA-damaging inducer, is especially harmful to photosynthetic organisms that are continuously exposed to sunlight [1, 2]. UV-B promotes covalent linkages between two adjacent pyrimidines, generating cyclobutane pyrimidine dimers (CPDs) or [6–4] pyrimidine–pyrimidone photoproducts ([6–4] PPs) [3]. These pyrimidine dimers can impede the progress of replicative DNA polymerases (DNAP), resulting in replication fork collapse that is potentially lethal to cells [4–8]. Diverse routes such as fork reversal, lesion skipping, and translesion DNA synthesis (TLS) are used by the cell to cope with pyrimidine dimers during cellular replication [7, 9–11]. In contrast to the nucleus [12–15], plant organelles display an incomplete set of UV lesion repair pathways: photolyases are present but enzymes for the nucleotide excision repair (NER) pathway are absent [16–18]. DNA replication in plant mitochondria and chloroplasts by organellar DNA polymerases (POPs), therefore, would occur on DNA templates containing CPDs or [6–4] PPs; nonetheless, replicative DNAPs in general lack TLS activity. POPs belong to the family A of DNA polymerases and function as organellar DNA replicases in plants and protists but are not present in fungi or animals [19–21]. POPs possess three insertions in their polymerization domain that are absent in replicative family A polymerases [20, 22, 23], which resemble the insertions present in human DNA repair polymerase Pol θ [24, 25]. These insertions in *Arabidopsis thaliana* POPs (AtPolIA and AtPolIB) are key components to their DNA repair activities, such as translesion synthesis across an abasic site or thymine glycols [23, 24, 26], dRP lyase, and microhomology-mediated end-joining (MMEJ) [23, 26–29].

Here we explored AtPolls’ ability to bypass CPD and [6–4] PPs lesions and evaluated the contribution of the three insertions to translesion synthesis activity. We found that AtPolIs efficiently bypass CPD using magnesium or manganese ions as cofactors, while [6–4] PP is only bypassed in the presence of Mn^2+^. Suppression of the modest exonuclease activity of AtPolls resulted in a significant increase in [6–4] PP bypassing synthesis. Deletion of each individual amino acid insertion drastically decreased their CPD bypassing activity, while [6–4] PP extension was completely abolished. The presence of manganese as a reaction component restores CPD but not [6–4] PP TLS activity.

## Materials and methods

### Plasmid constructs of AtPolIs

Optimized synthetic genes of *AtPolIA* (At1g50840) and *AtPolIB* (At3g20540) lacking the dual targeting sequence and the disordered region were subcloned into the *Nde* I and *BamH* I restriction sites of the modified pET19b vector containing a N-terminal 9× His tag and a PreScission protease cleavage site. AtPolIA and AtPolIB exonuclease-deficient mutants were designed as previously reported [23] using the Q5 Site-Directed Mutagenesis protocol (New England Biolabs, Ipswich, MA, USA). Constructs for ΔIns1, ΔIns2, and ΔIns3 were designed using background exo-deficient AtPolIB variant as described in a previous report [23].

### Protein expression and purification

Plasmids containing the different AtPolIs variants were overexpressed in *Escherichia coli* Rosetta 2 (DE3) Novagen^®^ (MilliporeSigma, Rockville, MD, USA). Bacterial cultures supplemented with 100 μg ml^−1^ of ampicillin were incubated at 37°C and 200 rpm to reach an OD_600_ of 0.5. Cultures were induced by adding 0.5 mM IPTG and incubated for 17 h at 17°C.

The different AtPolI variants were purified using the same protocol. Briefly, bacterial pellet was resuspended in a buffer containing 25 mM HEPES pH 8.0, 10% glycerol, 15 mM imidazole pH 8.0, 1 mM PMSF, 500 mM NaCl, and 0.2 mg ml^−1^ lysozyme. The resuspended solution was incubated on ice for 30 min before sonication. Lysate fraction was clarified at 30 000 × *g* for 60 min at 4°C. The supernatant was incubated with Qiagen Ni-NTA agarose resin (Qiagen, Germantown, MD, USA) for 70 min while rocking at 4°C. The nickel resin was washed twice with lysis buffer containing 30 and 50 mM of imidazole. The protein was eluted with lysis buffer containing 500 mM imidazole and dialyzed in 25 mM HEPES pH 8.0, 10% glycerol, 200 mM NaCl, 2 mM EDTA pH 8.0, 2 mM DTT. After dialysis, the protein fraction was concentrated and loaded onto a HiTrap heparin HP column (Cytiva, Marlborough, MA, USA). Bound protein was eluted using a NaCl gradient (50–1000 mM). Fractions with pure protein were pooled, concentrated, and loaded into a Superdex 16/60 200 column (Cytiva, Marlborough, MA, USA) and eluted using a buffer E (25 mM HEPES pH 8.0, 10% glycerol, 100 mM NaCl, 2 mM EDTA pH 8.0, 2 mM DTT). Pure fractions were pooled and concentrated in the gel filtration buffer and stored at −80°C until use.

### Oligonucleotide substrates

DNA oligonucleotides containing a CPD and a [6–4] PP were synthesized as previously described (Table 1) [30, 31]. An equivalent non-damaged DNA template (ND) and FAM-labeled primers were purchased from Integrated DNA Technologies (Table 1). We annealed the following dsDNA substrates: CPD/N, CPD/N + 1, CPD/N + 2, CPD/N + 3, [6–4] PP/N, [6–4] PP/N + 1, [6–4] PP/N + 1T, [6–4] PP/N + 1G, [6–4] PP/N + 2, [6–4] PP/N + 3, [6–4] PP/N + 5, ND/N, ND/N + 1, ND/N + 2, ND/N + 3, and ND/N + 5 by mixing the FAM-labeled primer with the complementary oligonucleotide, incubating at 95°C for 5 min followed by slow cooling to room temperature, and storing at −20°C.

**Table 1.** Name and sequences of DNA used in this study

Name	Sequence
Primer N	FAM 5′-AGC TAT GAC CAT GAT TAC GAA TTG CTT-3′
Primer N + 1	FAM 5′-AGC TAT GAC CAT GAT TAC GAA TTG CTT A-3′
Primer N + 2	FAM 5′-AGC TAT GAC CAT GAT TAC GAA TTG CTT AA-3′
Primer N + 3	FAM 5′-AGC TAT GAC CAT GAT TAC GAA TTG CTT AAT-3′
Primer N + 5	FAM 5′-AGC TAT GAC CAT GAT TAC GAA TTG CTT AAT TC-3′
Primer N + 1T	FAM 5′-AGC TAT GAC CAT GAT TAC GAA TTG CTT T-3′
Primer N + 1G	FAM 5′-AGC TAT GAC CAT GAT TAC GAA TTG CTT G-3′
ND	3'-TCG ATA CTG GTA CTA ATG CTT AAC GAA TTA AGC ACG TCC GTA CCA TCG A-5'
CPD	3'-TCG ATA CTG GTA CTA ATG CTT AAC GAA (CPD)A AGC ACG TCC GTA CCA TCG A-5'
[6–4] PP	3'-TCG ATA CTG GTA CTA ATG CTT AAC GAA ([6–4] PP) A AGC ACG TCC GTA CCA TCG A-5'

### Translesion DNA synthesis assay

We run translesion DNA synthesis assays to test AtPolIs exo+ and AtPolIs exo− variants. Reactions were performed in a buffer containing 10 mM Tris–HCl pH 7.7, 50 mM NaCl, 5% glycerol, 1.5 mM DTT, 0.2 mg ml^−1^ BSA with 100 nM of substrate ND/N, CPD/N, or [6–4] PP/N mixed with 50 or 200 nM of AtPolIA and AtPolIB variants. After 5 min of preincubation at 37°C, reactions were initiated by adding 200 μM of each dNTP and 2 mM of Mg^2+^ or 2 mM Mn^2+^. Reactions were quenched after 5 min by adding nine-fold excess of quenching buffer (80% formamide, 50 mM EDTA pH 8, 0.1% SDS, 5% glycerol, and 0.02% bromophenol blue). To test the TLS activity of the mutants ΔIns1, ΔIns2, and ΔIns3, we followed the same reaction conditions as described above but quenched reactions at different times.

In parallel, T7 DNAP exo+ was assayed where 100 nM of ND/N, CPD/N, or [6–4] PP/N substrate and 50 or 200 nM of enzyme were combined in a TR buffer (20 mM Tris–HCl pH 7.5, 1 mM DTT, 0.2 mg mL^−1^ BSA) as recommended by the manufacturer. Reactions were incubated under the same conditions as for AtPolIs but the catalysis buffer contained 10 mM of Mg^2+^ or Mn^2+^. Samples were boiled at 95°C for 5 min and resolved on a 17% polyacrylamide gel containing 8 M urea. Bands were visualized using FAM fluorescence on a GE Typhoon FLA 9000 Gel Scanner (Cytiva, Marlborough, MA).

### Exonuclease assays

To evaluate the exonuclease activity of the AtPollA and AtPolIB, we followed the same conditions for the translesion DNA synthesis assay as described above but the catalysis buffer did not contain dNTPs. We tested ND/N, ND/N + 1, ND/N + 2, ND/N + 3, and ND/N + 5 and the equivalent substrates containing CPD or [6–4] PP lesions. Samples were stopped at indicated times, boiled at 95°C for 5 min, and resolved on a 17% polyacrylamide gel containing 8 M urea. Gels were scanned in a GE Typhoon FLA 9000 Gel Scanner (Cytiva, Marlborough, MA).

### TLS activity under divalent metal ion titration

To characterize the influence of different Mg^2+^ and Mn^2+^ concentrations on the AtPolls activities, we performed DNA extension assays by combining 100 nM of DNA substrate (ND/PN, CPD/PN, [6–4] PP/CPD/PN) with 200 nM of AtPolIA exo+ or AtPolIB exo+. The reactions started by adding 0.2 mM dNTPs and a gradient of 0–10 mM of Mg^2+^ or Mn^2+^. After 5 min of incubation at 37°C, samples were quenched by adding nine-fold excess of quenching buffer (80% formamide, 50 mM EDTA pH 8, 0.1% SDS, 5% glycerol, and 0.02% bromophenol blue). Samples were resolved on a 17% polyacrylamide gel containing 8 M urea and scanned on a GE Typhoon FLA 9000 Gel Scanner (Cytiva, Marlborough, MA).

### DNA extension of UV lesion-containing substrates

To evaluate whether AtPolIs can extend intermediate product mimicking stages of CPD and [6–4] PP, we used the dsDNA substrates CPD/N, CPD/N + 1, CPD/N + 2, CPD/N + 3, [6–4] PP/N, [6–4] PP/N + 1, [6–4] PP/N + 2, and [6–4] PP/N + 3. The 3′ OH end of the primer N is right before the lesion, N + 1 covers 3′-T of the lesion, N + 2 the whole lesion, and N + 3 pairs one base after the lesion. One hundred nanomolar DNA and 200 nM of DNA polymerase (AtPolIA exo+, AtPolIA exo−, AtPolIB exo+, AtPolIB exo−) were mixed in 10 mM Tris–HCl pH 7.7, 50 mM NaCl, 5% glycerol, 1.5 mM DTT, 0.2 mg ml^−1^ BSA. Reactions were preincubated at 37°C for 5 min and started by addition of 0.2 mM of each dNTP and 2 mM of Mg^2+^ or 2 mM Mn^2+^. As a control, we run reactions by combining 100 nM of the equivalent non-damaged DNA (ND) with 200 nM of DNA polymerase. Reactions were stopped at 5 min by the addition of nine-fold excess of quenching buffer (80% formamide, 50 mM EDTA pH 8, 0.1% SDS, 5% glycerol, and 0.02% bromophenol blue). Quenched samples were boiled at 95°C for 5 min and resolved on a 17% polyacrylamide gel containing 8 M urea. Gels were visualized using FAM-fluorescence in a GE Typhoon FLA 9000 Gel Scanner (Cytiva, Marlborough, MA).

### Single nucleotide incorporation

To determine the preferential nucleotide incorporation opposite CPD and [6–4] PP, we used the dsDNA substrates CPD/N, CPD/N + 1, CPD/N + 2, CPD/N + 3, [6–4] PP/N, [6–4] PP/N + 1, [6–4] PP/N + 1T, [6–4] PP/N + 1G, [6–4] PP/N + 2 and [6–4] PP/N + 3, and their equivalent ND substrates. One hundred nanomolar DNA substrate and 200 nM of AtPolIA exo− or AtPolIB exo− were preincubated at 37°C in a buffer containing 10 mM Tris–HCl pH 7.7, 50 mM NaCl, 5% glycerol, 1.5 mM DTT, 0.2 mg ml^−1^ BSA. Reactions started with the addition of 2 mM Mg^2+^ and 2.5 μM of dATP, dTTP, dGTP, or dCTP (for ND), 100 μM for CPD, and 400 μM for [6–4] PP. All samples were stopped by the addition of nine-fold excess quenching buffer (80% formamide, 50 mM EDTA pH 8, 0.1% SDS, 5% glycerol, and 0.02% bromophenol blue) after an incubation period of 1 min at 37°C, with the exception of reactions containing [6–4] PP/PN + 1, which were stopped after 5 min to ensure incorporation. Quenched samples were boiled at 95°C for 5 min and resolved on a 20% polyacrylamide gel containing 8 M urea. Gels were scanned using a GE Typhoon FLA 9000 Gel Scanner (Cytiva, Marlborough, MA).

### EMSA DNA-binding assays

DNA binding affinity of AtPolIB and insertion mutants to a normal and damaged DNA template was determined using an EMSA approach. Ten nanomolar of ND/N, CPD/N, or [6–4] PP/N template and the indicated enzyme concentrations were mixed in a buffer containing 10 mM Tris–HCl pH 7.7, 50 mM NaCl, 10% glycerol, 1.5 mM DTT, 0.2 mg ml^−1^ BSA and incubated at room temperature for 15 min. The mixtures were directly loaded on a 6% native polyacrylamide gel in a 0.5× TBE for 45 min at 180 V at 4°C. Gels were visualized by fluorescence in a GE Typhoon FLA 9000 Gel Scanner (Cytiva, Marlborough, MA).

### Quantification and analysis

Scanned gels were analyzed using ImageQuant TL (GE Healthcare). First, we subtracted the background following the rolling ball method. For primer extension plots, the percentage of full-length products was calculated using the ratio of full-length extension band intensity to the sum of the total band intensities in each lane. The rate of excision (*k*_exo_) of the 3′-end of the different primers was determined by plotting the consumption of the primer substrate against time and fitting the data to a one-phase decay model using GraphPad Prism (version 10.6). The observed polymerization rate (*K*_obs_) was determined by plotting the product band versus time and fitted to the four-parameter logistic (nonlinear regression) using GraphPad Prism (version 10.6).

For DNA binding dissociation constant. Band intensities corresponding to unbound and bound DNA were quantified using the ImageQuant software. Background-corrected intensities were used to determine bound DNA/(unbound DNA + bound DNA) fractions. The bound DNA fraction was plotted against the enzyme concentration and fitted to a non-linear regression in GraphPad Prism (version 10.6).

## Results

### AtPolIs displays TLS activity across CPD and [6–4] PP

**Figure 1.**
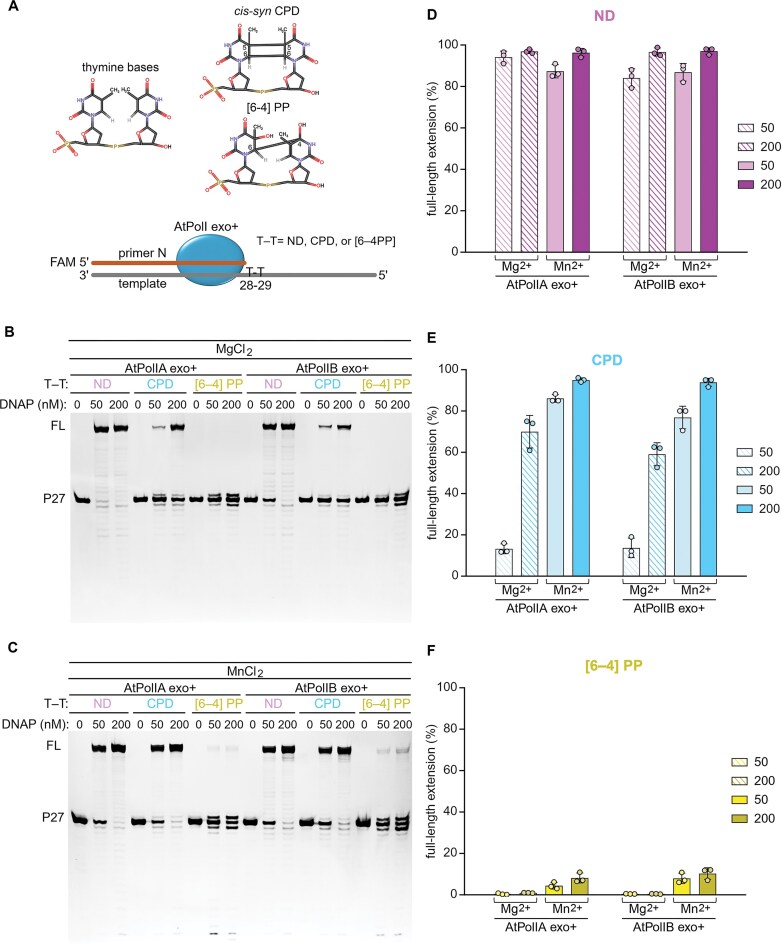
Translesion DNA synthesis assays on CPD and [6–4] PP by AtPolls exo+. (**A**) Schematic representation of a CPD and a [6–4] PP (*top*). Cartoon depicting the DNA substrate used for lesion bypassing assays (*bottom*). (**B**) 17% denaturing gel displaying lesion bypassing assays where 50 or 200 nM of AtPolIA exo+ or AtPolIB exo+ were combined with 100 nM of DNA substrate (CPD, [6–4] PP, or ND) in the presence of 2.0 mM Mg^2+^. (**C**) primer extension as in panel B but using 2.0 mM Mn^2+^. (**D**–**F**) Plots represent the percentage of extended primer for non-damaged (magenta), CPD (cyan), and [6–4] PP (yellow). Error bars represent the standard deviation from three repeats.

In previous works, we showed that AtPolIs from *Arabidopsis thaliana* bypass DNA lesions such as thymine glycols and abasic sites [23, 26]. To characterize the ability of these plant organellar DNAPs to bypass more detrimental and voluminous DNA damages, we examined their TLS activity on two UV lesions, CPD and [6–4] PP, in the presence of Mg^2+^ or Mn^2+^ on a substrate formed by a 49-nt DNA template containing a CPD or [6–4] PP and a 27-nt primer with the 3ꞌ-OH end right before the lesion (Fig. 1A). A non-damaged DNA substrate (ND) was assembled with identical sequence except for replacing the lesion with two thymines. We performed the assays using sub-stoichiometric and two-fold excess enzyme concentrations to determine the optimal conditions under which TLS appears. In the presence of 2 mM Mg^2+^, AtPolIA and AtPolIB extended 75% and 62% of the CPD substrate, respectively (Fig. 1B and E), demonstrating substantial capacity for CPD lesion bypass. In contrast, AtPolls incorporated only a single nucleotide on the [6–4] PP without further extension (Fig. 1F and B). Interestingly, when the assays were repeated using 2 mM Mn²⁺, the overall TLS activities are markedly enhanced. The highest Mn^2+^ concentration influence on CPD extension was observed in the condition with 50 nM of enzyme, where the product formation increased from 12% to 85% (AtPolIA exo+) and 13% to 80% (AtPolIB exo+) compared to reactions containing Mg^2+^ (Fig. 1C  *left* and Fig. 1E  *left*). On the [6–4] PP template, AtPolIA and AtPolIB extended ∼8% and ∼11%, respectively (Fig. 1C and F), whereas both AtPolls extended nearly 100% of the primer to full-length on non-damaged (ND) template in the presence of either Mg^2+^ or Mn^2+^ (Fig. 1D). Although the bypass efficiency was modest, these results suggest that AtPolls would be the only replicative DNA polymerase reported with intrinsic [6–4] PP TLS activity. We next assayed T7 DNA polymerase (T7 DNAP), a DNAP unable to bypass UV lesions, to evaluate whether Mn²⁺ can stimulate lesion bypass in other replicative DNA polymerases. No bypass products were detected on either the CPD or [6–4] PP templates (Supplementary Fig. S1B). Instead, Mn²⁺ drastically reduced T7 DNAP activity on the ND template, consistent with previous reports [32] (Supplementary Fig. S1C).

### AtPolIs displays modest exonuclease activity

DNAPs with a functional exonuclease domain preferentially move the DNA toward the 3′–5′-exonuclease (exo) site for degradation, preventing further lesion extension and negatively regulating TLS activity. To evaluate whether the intermediate products during UV lesion bypassing are detected in comparison to normal substrates, we determined excision rates for CPD and [6–4] PP annealed to different primer lengths (N, N + 1, N + 3, N + 5). Our results indicate that AtPolIA displayed an ∼1.6-fold higher excision rate than AtPolIB on undamaged DNA (Table 2 and Supplementary Fig. S2), which correlates with previous reports where AtPolIA exhibits higher fidelity and reduced error rates [33]. We found that AtPolIA increased the excision rates by 1.3 to 3-fold, while AtPolIB increased the excision rates between 2.1 and 3.8-fold in substrates containing the UV lesion compared to undamaged DNA (Table 2 and Supplementary Fig. S2). Despite the increase in excision rates for AtPolIB on lesioned DNA compared to normal substrates, AtPolIA displays only a slight increment of excision rate on damaged substrates. When we compared excision rates for DNA substrates using primers paired before, at, and after the lesion, both AtPolIs increased lesion detection on substrates where the primer is located opposite the lesion and one position after it. While the excision rates were reduced when the primer was behind or 3 positions after the UV lesion (Table 2 and Supplementary Fig. S2). These results suggest that AtPolIs possesses an exonuclease activity that moderately detects CPD and [6–4] PP lesions when the primer terminus is positioned opposite the lesion, whereas moving the primer a few nucleotides beyond reduces the lesion recognition ability. This feature would be one of the factors triggering the strong TLS activity of AtPolIs.

**Table 2.** Exonuclease rate of AtPolIA exo+ and AtPolIB exo+ on ND, CPD, and [6–4] PP substrates

template	primer	AtPolIA exo+		AtPolIB exo+	
		*k*_obs_ (*s^-1^*)	FC*^a^*	*k*_obs_ (*s^-1^*)	FC*^b^*
ND	N	0.035 ± 0.01	-	0.019 ± 0.004	-
	N + 1	0.047 ± 0.006	-	0.029 ± 0.005	-
	N + 2	0.036 ± 0.013	-	0.032 ± 0.007	-
	N + 3	0.039 ± 0.014	-	0.026 ± 0.006	-
	N + 5	0.042 ± 0.007	-	0.022 ± 0.005	-
CPD	N	0.045 ± 0.011	1.3	0.039 ± 0.01	2.1
	N + 1	0.102 ± 0.012	2.2	0.091 ± 0.018	3.1
	N + 2	0.089 ± 0.008	2.5	0.077 ± 0.013	2.4
	N + 3	0.106 ± 0.017	2.7	0.096 ± 0.024	3.7
	N + 5	0.057 ± 0.010	1.4	0.047 ± 0.010	2.1
[6–4] PP	N	0.050 ± 0.005	1.4	0.046 ± 0.009	2.4
	N + 1	0.098 ± 0.012	2.1	0.089 ± 0.018	3.1
	N + 2	0.092 ± 0.01	2.6	0.085 ± 0.012	2.7
	N + 3	0.115 ± 0.013	3.0	0.098 ± 0.015	3.8
	N + 5	0.055 ± 0.006	1.3	0.054 ± 0.011	2.5

FC indicates fold-change increment of AtPolIA (*a*) and AtPolIB (*b*) relative to their normal DNA values.

### Optimal metal ion concentrations for TLS activity of AtPolIs

**Figure 2.**
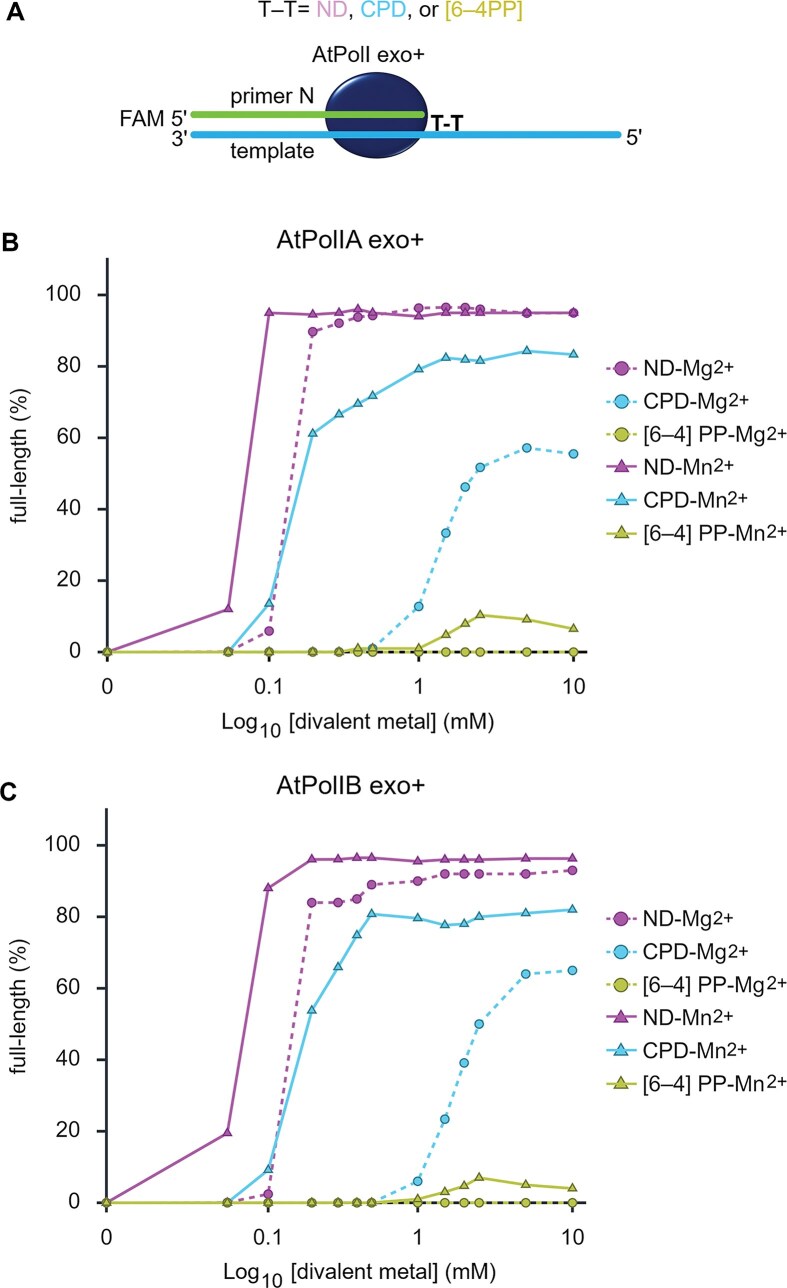
AtPolIs translesion DNA synthesis of exo-proficient AtPolIs under an Mg^2+^ and Mn^2+^ gradients. (**A**) Design of substrates using a primer N (27-nt) with the 3′-end placed right before the CPD, [6–4] PP, or normal template. (**B**) Percentage of full-length product plotted against 0–10 mM of Mg^2+^ or Mn^2+^. AtPolIA exo+ displays an overall increment of product in the presence of Mn^2+^ (solid line) from lower concentration than assays containing Mg^2+^ (dotted line). (**C**) As in panel (B) but using AtPolIB exo+. ND and CPD product formation increased in the presence of Mn^2+^ compared to Mg^2+^. As in panel (B), AtPolIB exo+ full extension begins at lower Mn^2+^ concentration. Plots were constructed using gels from Supplementary Fig. S3.

As AtPolIs displayed metal-dependent TLS, we investigated the optimal metal ion concentration that supports AtPolIs TLS activity. Assays were conducted from 0.05 to 10 mM Mg^2+^ or Mn^2+^. On ND template, AtPolIA exo+ displayed full extension products from 0.2 to 10 mM Mg^2+^ (Fig. 2B and Supplementary Fig. S3A  *left*), whereas on the CPD-containing template, the full-length (FL) product was observed from 1 to 10 mM Mg^2+^ reaching a plateau from 2.5 to 10 mM (Fig. 2B and Supplementary Fig. S3A  *middle*). In contrast to ND reactions, where exonucleolytic products are barely detectable, on CPD templates the exonucleolytic products increased proportionally with the Mg^2+^ concentration, while full-length products remained almost the same (2.5–10 mM) (Fig. 2B and Supplementary Fig. S3A  *middle* compared to S3A *left*). On [6–4] PP substrates, AtPollA exo+ was unable to extend the substrate, and only a slight exonuclease activity was observed from 1.5 to 10 mM Mg^2+^ (Supplementary Fig. S3A  *right*). Inspired by previous investigations showing that Mn^2+^ can stimulate bypassing abilities on other DNAPs, we repeated primer extension in the presence of different Mn^2+^ concentrations. AtPolIA exo+ extended ND substrate at lower concentration (0.05 mM) than in the presence of Mg^2+^ (Fig. 2A and Supplementary Fig. 3B  *left* compared to Supplementary Fig. S3A  *left*), consistent with previous investigations showing that Mn^2+^ binds to the Pol site with higher affinity than Mg^2+^ [32]. On the CPD template, the FL product began at 0.1 mM and reached the maximum ∼80% extension at 0.4 to 0.5 mM Mn^2+^, and simultaneously, an elevated exonuclease activity was observed from 1 to 10 mM Mn^2+^ (Fig. 2B and Supplementary Fig. 3B  *middle*). On the [6–4] PP template, AtPolIA exo+ extended ~11% at 2.0–5.0 mM Mn^2+^ (Supplementary Fig. S3B  *right*). Although the exonucleolytic pattern is similar to that on the CPD template, the unextended intermediates (28- and 29-nt) and the primer substrate (27-nt) in [6–4] PP reactions are not further digested (Supplementary Fig. S3B  *middle* compared to S3B *right*). The latter suggests that AtPolIA exo+ would enter a treadmilling cycle, where nucleotides are incorporated and removed, preventing a complete primer degradation.

AtPolIB exo+ exhibited similar metal-dependent activities on ND, CPD, and [6–4] PP as AtPolIA exo+. Full primer extension product on ND template was observed from 0.2 to 10 mM Mg^2+^; on CPD-template, from 1 to 10 mM Mg^2+^ and no bypassing activity on the [6–4] PP template (Fig. 2C). However, we noticed that AtPolIB exo+ generated longer exonucleolytic products than AtPolIA exo+ on the CPD templates (Supplementary Fig. S3C  *middle* compared to Supplementary Fig. S3A  *middle*), which correlates with the lower excision rates in AtPolIB than AtPolIA for normal and damaged substrates.

In the presence of Mn^2+^, AtPolIB exo+ showed CPD TLS activity in a wider metal range (0.1–10 mM), reaching 90% FL products from 1–10 mM Mn^2+^ (Fig. 2C and Supplementary Fig. S3D  *middle*). On [6–4] PP reactions, Mn^2+^ triggered a limited extension of ~10% (1.5–5 mM) (Fig. 2C and Supplementary Fig. S3D  *right*). Overall, these results suggest a correlation between the metal ion concentration, the exonuclease, and the TLS activity. In addition, our findings indicate an earlier divalent metal saturation of the polymerase over exonuclease active site, which would favor bypassing activity before primer degradation.

Furthermore, using a mixture of Mg^2+^ and Mn^2+^ metal ions, we found that both AtPolIs exo+ can also extend CPD and [6–4] PP lesions (Supplementary Fig. S4), suggesting that these DNAPs are capable of using Mn^2+^ in the presence of Mg^2+^, which supports the idea that TLS on [6–4] PP would be feasible in cells where both divalent metals are present.

### Extension of intermediates CPD and [6–4] PP by wild-type AtPolIs

**Figure 3.**
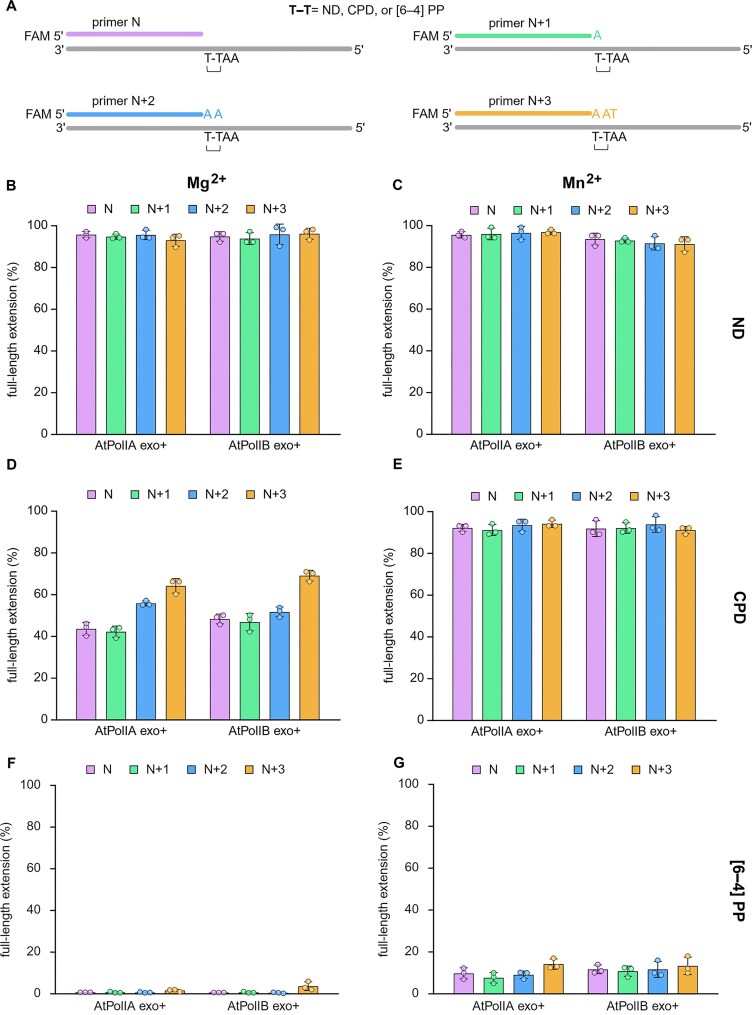
Primer extension of UV lesion-containing DNA in the presence of Mg^2+^ or Mn^2+^ by exo-proficient AtPolIs. (**A**) Scheme of the four DNA substrates used for each template (ND, CPD, or [6–4] PP), where the FAM-labeled primer increases by 1 nt. Plot indicating the full extension by AtPolIA exo+ or AtPolIB exo+ incubated with four different non-damaged substrates (N, N1, N2, N3, N4) in the presence of 2 mM Mg^2+^ (**B**) or 2 mM Mn^2+^ (**C**). Extension of four CPD substrates by AtPolIA exo or AtPolIB exo+ using 2 mM Mg^2+^ (**D**) or 2 mM Mn^2+^ (**E**). As in panels (D, E) but using four substrates containing a [6–4] PP lesion in the presence of 2 mM Mg^2+^ (**F**) or 2 mM Mn^2+^ (**G**). Error bars represent the standard deviation from three repeats.

To investigate whether exonuclease-proficient AtPolIs can extend stalled products during CPD and [6–4] PP replication, we designed primers that mimic sequential bypassing of a UV lesion at 1-nt increments, termed N, N + 1, N + 2, and N + 3 (Fig. 3A). Both AtPolIs extended all four ND templates with the same efficiency (Fig. 3B and C and Supplementary Fig. S5B  *left* and D *left*).

On the CPD template and with 2 mM Mg^2+^, AtPolIA exo+ fully extended ∼45% of the N and N + 1 primer, 57% of N + 2, and 65% of N + 3 primer (Fig. 3D and Supplementary Fig. S5B  *middle*). While in the presence of 2 mM Mn^2+^, 90% of all four primers were fully extended (Fig. 3E and Supplementary Fig. S5D  *middle*). AtPoIIB exo+ extended the CPD template with similar efficiency to AtPolIA exo+, displaying the highest FL product formation on the substrate with the primer N + 3 located one base after the lesion (Fig. 3D and Supplementary Fig. S5C  *middle*). As observed for AtPolIA exo+ reactions, in the presence of 2 mM Mn^2+^ AtPolIB exo+ displayed 90% of extension products on the four CPD templates (Fig. 3E and Supplementary Fig. S5D  *middle*). Overall, these results suggest that the primer length is positively correlated with the ability of AtPolIs exo+ trans-CPD lesion extension in the presence of Mg^2+^, whereas Mn^2+^ stimulates all primer trans-CPD extension uniformly.

On the [6–4] PP templates, both AtPolIs were unable to extend any template with 2 mM Mg^2+^ (Fig. 3F and Supplementary Fig. S5C  *right*). In the presence of 2 mM Mn^2+^, AtPolIA exo+ and AtPoIIB extended 8%–14% and 10%–13% of the primers N, N + 1, N + 2, and N + 3, respectively (Fig. 3G), which is modestly increased in comparison to reactions containing Mg^2+^ (Fig. 3F compared to Fig. 3G).

One interesting observation is that in reactions containing Mg^2+^, the primer leftover for all CPD and [6–4] PP substrates (N, N + 1, N + 2, N + 3) resemble the N primer length (27-nt) instead of 28, 29, or 30 nt for N + 1, N + 2, and N + 3 primers, respectively (Supplementary Fig. S5B and C). This indicates that AtPolIs exo+ still detects the UV lesion even one position after the lesion (N + 3), triggering an excision activity that preferentially stops when the 3′-OH end of the primer is located right before the lesion. These results suggest that AtPolls generate at least two groups of extended primers, one group treadmills at the lesion site where one nucleotide incorporation and excision occur alternatively, and another group of primers is fully extended. Interestingly, AtPolIB exo+ displays a less efficient cleaving ability on the [6–4] PP templates consistent with its lower exonuclease activity compared to AtPolIA exo+ (Supplementary Fig. S5C  *right*).

**Figure 4.**
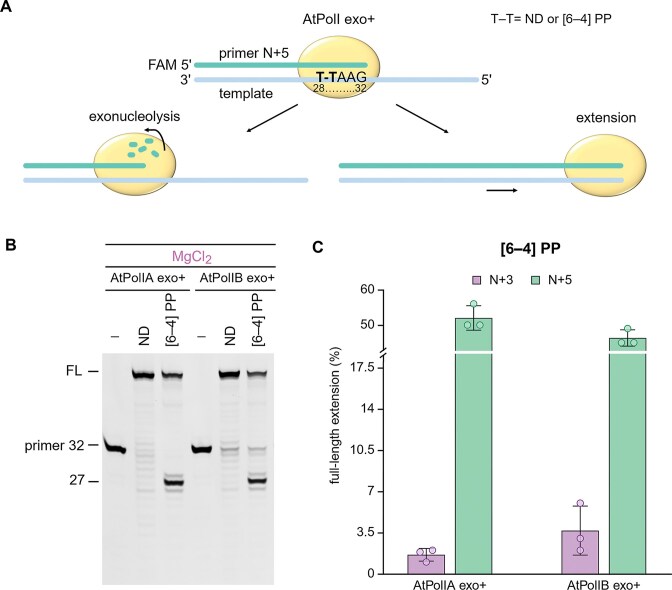
[6–4] PP extension by AtPolIs exo+ in the presence of Mg^2+^. (**A**) Cartoon of the DNA substrate where the primer is located at the +3 position after the lesion. The scheme shows the two possible mechanisms when AtPolIs exo+ detects a bypassed lesion. (**B**) Extension assay of a non-damaged (ND) or [6–4] PP template in the presence of 2 mM Mg^2+^. FL indicates full-length product; primer substrate is shown as a 32-nt band, and the exonucleolytic product as 27-nt (right before the [6–4] PP lesion position). (**C**) Plot indicating the full-length extension by AtPolIA exo and AtPolIB exo+ on the [6–4] PP/N + 3 (replotted from Fig. 3F) and [6–4] PP/N + 5 (from panel B) in the presence of 2 mM Mg^2+^. Error bars represent the standard deviation from three repeats.

To address this lesion-detection ability, we used a 32 nt primer (N + 5) where the 3′-end is +3 positions past the [6–4] PP lesion (Fig. 4A). In the presence of Mg^2+^, both AtPolIs significantly increased the [6–4] PP extension efficiency (Fig. 4). These values represent a 25-fold and 12-fold increase in [6–4] PP extension by AtPolIA exo+ and AtPolIB exo+, respectively, compared to extension of primer N + 3 in the presence of Mg^2+^ (Fig. 4C). These findings suggest that AtPolIs can sense the lesion a couple of positions after and reduce as polymerization progresses, which correlates with the reduced excision rate on the same [6–4] PP/N + 5 substrate (Supplementary Fig. S2).

### Mn^2+^ enables robust [6–4] PP TLS by exo-deficient AtPolIs

**Figure 5.**
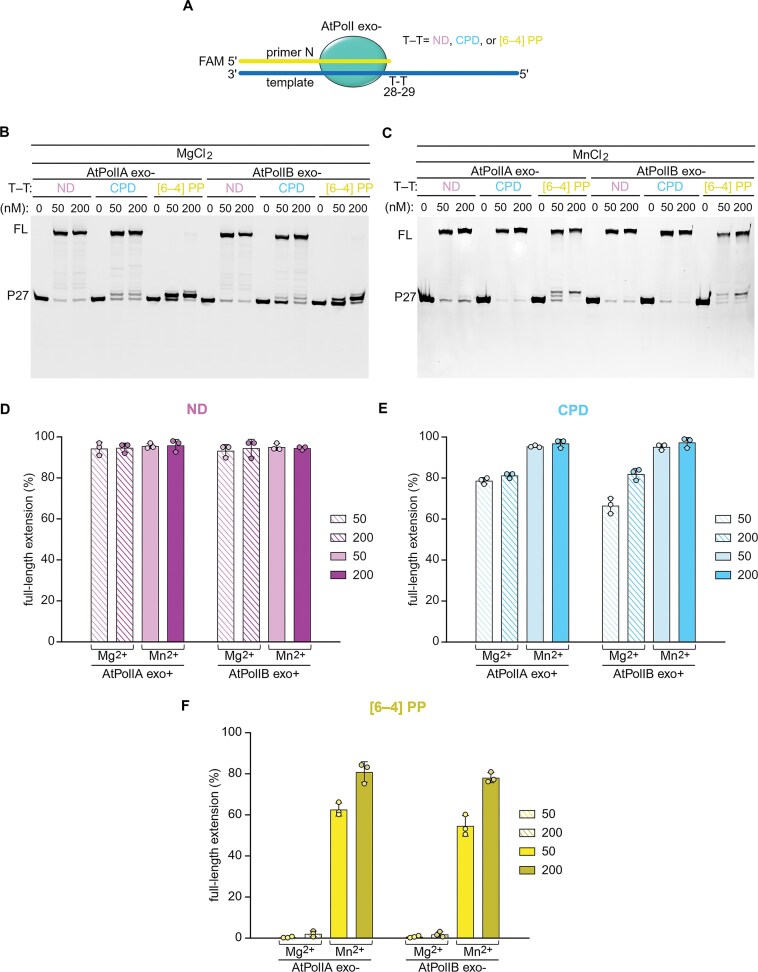
Lesion bypassing assays on CPD and [6–4] PP by exo-deficient AtPolIs. (**A**) DNA substrates used in this assay were constituted by a 27-nt FAM-labeled primer and a 49-nt template. T-T denotes a CPD, [6–4] PP, or two adjacent thymines. (**B**) Lesion bypassing assays where 50 or 200 nM of AtPolIA exo− or AtPolIB exo− were combined with 100 nM of DNA substrate in the presence of 2.0 mM Mg^2+^. (**C**) Primer extension as in panel B but using 2.0 mM Mn^2+^. Bar chart from panels (B, C), indicating the percentage of extended primer for non-damaged (**D**), CPD (**E**), and [6–4] PP (**F**) substrates. Error bars represent standard deviation from three repeats.

In a previous report, we demonstrated that AtPolIs display moderate exonuclease activity, resulting in a lower fidelity compared to human mitochondrial replicase Pol γ and T7 DNAP, as a trade-off for their ability to bypass DNA lesions [23, 33]. As the inactivation of the exonuclease domain in DNAPs potentiates at different extents their intrinsic TLS activities [23, 34, 35, 36], we studied the TLS activity on UV lesions using AtPolIs exo− variants. We tested the same CPD, [6–4] PP, and a non-damaged template substrate used for the AtPolIs exo+ (Fig. 5A). AtPolIA exo− and AtPolIB exo− fully extended the non-damaged (ND) substrate (95%) in the presence of either 2 mM of Mg^2+^ or 2 mM of Mn^2+^ (Fig. 5B–D). In reactions incubated with 2 mM of Mg^2+^, AtPolIB exo− showed 65 to 82% of CPD extension, while the presence of 2 mM Mn^2+^ increased the yield up to 95% (Fig. 5B, C, and E). AtPolIA exo− extended 77% to 82% of the CPD substrate when incubated with 2 mM Mg^2+^ and over 95% in a reaction containing 2 mM Mn^2+^ (Fig. 5B and E). In [6–4] PP reactions incubated with 2 mM Mg^2+^, both AtPolIs exo− variants did not exhibit extension products (Fig. 5B and F). While in reactions incubated with 2 mM Mn^2+^ both AtPolIs exo− show significant increase of [6–4] PP extension (Fig. 5). AtPolIB exo− extended 57% to 76%, while AtPolIA exo− yielded 62%–80% of products (Fig. 5C and F). Overall, diminishing the exonuclease function enhanced the TLS activity on AtPolIs. In CPD reactions containing 2 mM Mg^2+^, AtPolIA exo− and AtPolIB exo− increased the lesion-bypassing ability up to five- and four-fold, respectively, compared to their exonuclease-proficient counterparts (Fig. 1E compared to Fig. 5E, *see 50 nM conditions*). In [6–4] PP reactions containing 2 mM Mn^2+^, AtPolIB exo− enhanced the extension efficiency by seven-fold, while AtPolIA exo− displayed up to a 10-fold increment (Fig. 1F compared to 5F; *see 50 nM conditions*).

### Exo-deficient AtPolIs extend [6–4] PP DNA intermediates with high efficiency

**Figure 6.**
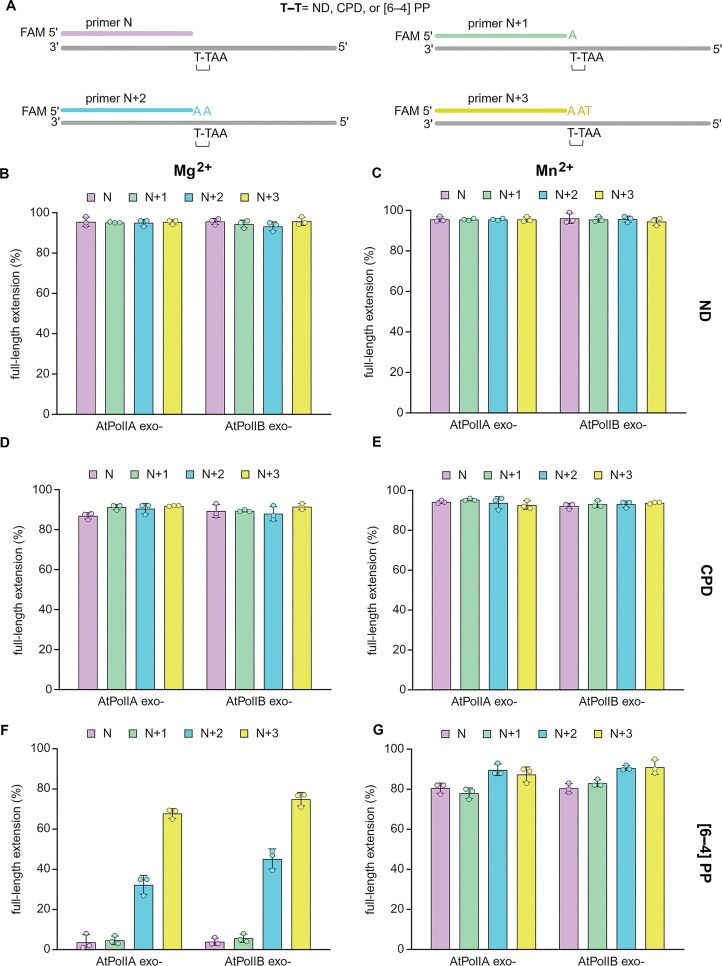
UV lesion extensions in the presence of Mg^2+^ or Mn^2+^ by exo-deficient AtPolIs. (**A**) Scheme of the twelve DNA substrates used in this assay. FAM-labeled primer varies by 1 nt: 27 (N), 28 (N + 1), 29 (N + 2), and 30 (N + 3). T-T denotes two thymines, CPD or [6–4] PP. Data from primer extension assays by AtPolIs exo− variants in the presence of 2.0 mM Mg^2+^ (**B**) or 2.0 mM Mn^2+^ (**C**). Data from bypassing assays using CPD substrates incubated with 2 mM Mg^2+^ (**D**) or 2 mM Mn^2+^ (**E**). AtPolIA exo− and AtPolIB exo− display increasing product formation from N to N + 3, ranging from 5% to 70% in the presence of 2 mM Mg^2+^ (**F**), while reactions incubated with 2.0 mM Mn^2+^ drastically increased the full extension products (**G**). Error bars represent the standard deviation from three repeats.

We performed extension assays for AtPolIs exo− variants using primers that mimic progressive bypassing of an UV lesion (Fig. 6A). In a CPD-containing substrate incubated with 2 mM Mg^2+^, AtPolIB exo− fully extended ∼90% of primers N, N1, N + 2, and N + 3 and greater than 95% in the presence of 2 mM Mn^2+^ (Fig. 6D and E, Supplementary Fig. S6C–E). All four ND templates were also extended with similar efficiency in the presence of either metal ions (Fig. 6A–C and Supplementary Fig. S6C–E). AtPolIA exo− presented similar extension for both ND and CPD templates (Fig. 6B–E and Supplementary Fig. S6B and D). In [6–4] PP samples, AtPolIB exo− exhibits limited extension for primers N and N + 1 with a significant increase for N + 2 and N + 3 when reactions were incubated with 2 mM Mg^2+^ (Fig. 6F and Supplementary Fig. S6C). Mn^2+^ enabled a robust [6–4] PP bypassing activity on AtPolIB exo− to extend the four templates with product yield ranging from 80% to 91% (Fig. 6F compared to 6G and Supplementary Fig. S6E). AtPolIA exo− was also able to extend the four [6–4] PP substrates in the presence of Mg^2+^, ranging from 5% to 70% (N to N + 3 primers), while the incubation with 2 mM Mn^2+^ improved the extension ability up to 90% with similar efficiency across the four primers (Fig. 6F and G and Supplementary Fig. S6B and D). These results suggest that AtPolIs can extend bypassed CPD or [6–4] PP intermediates even in the presence of Mg^2+^, while Mn^2+^ triggers a more robust intrinsic TLS ability, indicating that the exonuclease activity negatively modulates this function.

### Nucleotide preference of AtPolIs TLS activity on CPD and [6–4] PP

In the above results of the intermediates extension assay, we found that in the presence of 2 mM Mg^2+^, both AtPolIs exo− failed to extend [6–4] PP when it is paired to N and N + 1 primers, but could insert a single nucleotide (+1) opposite the 3′-T of the [6–4] PP (Supplementary Fig. S6B and C), suggesting that incorporation opposite the 5′-T of the [6–4] PP constitutes an energy barrier for further DNA extension.

**Figure 7.**
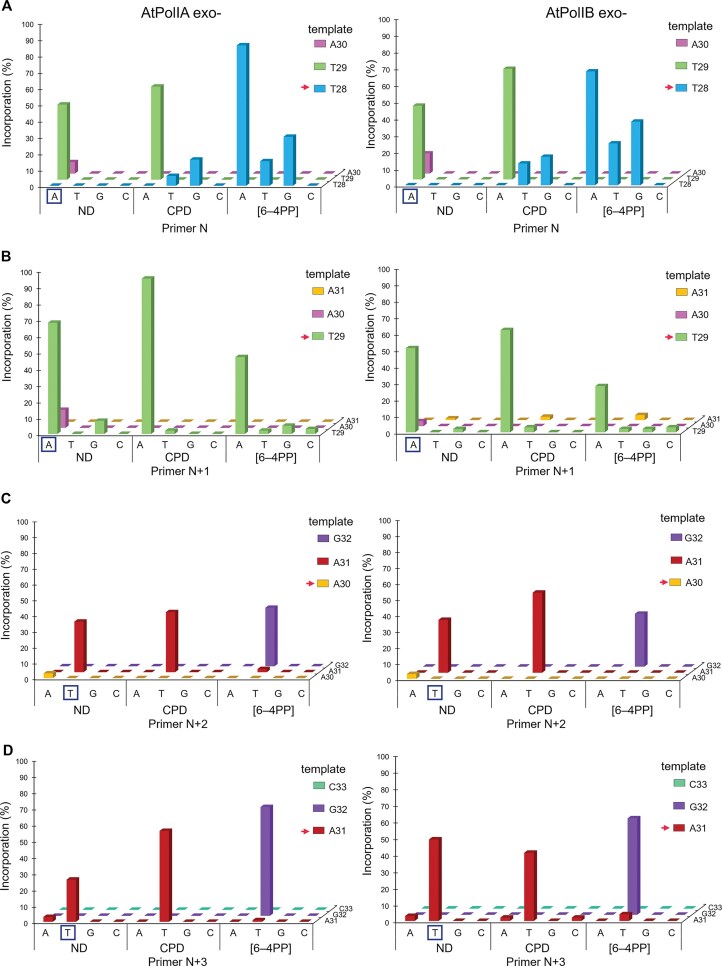
Single nucleotide incorporation opposite UV DNA lesions by AtPolIB exo−. Graphical representation of AtPolIA exo− and AtPolIB exo− single nucleotide incorporation on ND, CPD, and [6–4] PP templates and four different primer lengths. (**A**) For primer N, the first incorporation is at T28. On ND and CPD, two expected AMPs are incorporated while only one opposite [6–4] PP. (**B**) For primer N + 1, the template is a T29 where both AtPolIs exo− inserted a single dAMP on all substrates. (**C**) For primer N + 2, the first incoming nucleotide is at A30, where two expected dTMPs are inserted on ND and CPD, while for [6–4] PP and extra dTMP misinsertion is observed. (**D**) For primer N + 3, the insertion is at A31 template, and AtPolIs exo− inserted a single dTMP on ND and CPD templates while two dTMPs are incorporated on [6–4] PP. Red arrow indicates the templating base and blue box the expected nucleotide to be incorporated.

We first examined AtPolIs exo− primer extension from across lesions 3′-T (position N), 5′-T (N + 1), and after lesion at N + 2 and N + 3, assuming each prior incorporation is correct (Supplementary Fig. S7A). On the CPD-containing template, AtPolls primarily inserted correct nucleotides at all positions (Fig. 7A–D *middle*; Supplementary Fig. S7B–I  *middle*), with only minor misincorporation of dTMP and dGMP opposite of the 3′-T of CPD (Fig. 7A and Supplementary Fig. S7B and C *middle*), indicating a large error-free translesion synthesis. Contrarily, on the [6–4] PP/N template, both AtPolIs exo− could only insert a single dAMP, dTMP, or dGMP opposite to the 3′-T, but failed to extend any of them across 5′-T (Fig. 7A and Supplementary Fig. S7B and C  *middle*). Interestingly, AtPolls could insert a dAMP opposite to 5′-T from the primer N + 1 with a 3′-terminal “A” (Fig. 7B and Supplementary Fig. S7D and E *right*). Subsequent synthesis on [6–4] PP using N + 2 and N + 3 primers appeared to be correct incorporation and a G-T mismatch (Fig. 7C and D and Supplementary Fig. S7F–I).

Because TLS across [6–4] PP is more error-prone, we next analyzed the TLS from a primer containing an incorrectly incorporated nucleotide (Supplementary Fig. S8A). On the [6–4] PP-containing template, AtPolls exo− synthesis displayed an interesting pattern: if a correct dAMP is inserted opposite to 3′-T, then continuous incorporation opposite to 5′-T is error-free; conversely, if an incorrect dTTP is inserted opposite to 3′-T, the next incorporation is also an incorrect dCTP (Supplementary Fig. S8B and C *left* and *middle*). Misincorporation of a dGTP opposite to 3′-T-terminated TLS at the 5′-T position (Supplementary Fig. S8B and C *right*).

On the ND template, both AtPolIs exo− incorporated the correct dAMPs and dTMPs opposite the templating bases T28, T29, A30, and A31, respectively (Fig. 7 and Supplementary Fig. S7B–I  *left*).

These results indicate that, after a T-T mismatch in the first thymine of the [6–4] PP, AtPolIs will tend to insert a less voluminous dCMP base to reduce DNA distortion, ensuring further extension, while T-G mismatches result in a non-productive intermediate. Overall, our findings indicate that both AtPolIs execute a moderate error-free TLS on CPD, while on a [6–4] PP they follow an error-prone TLS pathway (Fig. 10C).

### TLS on AtPolIs reside on unique insertions at the polymerization domain

**Figure 8.**
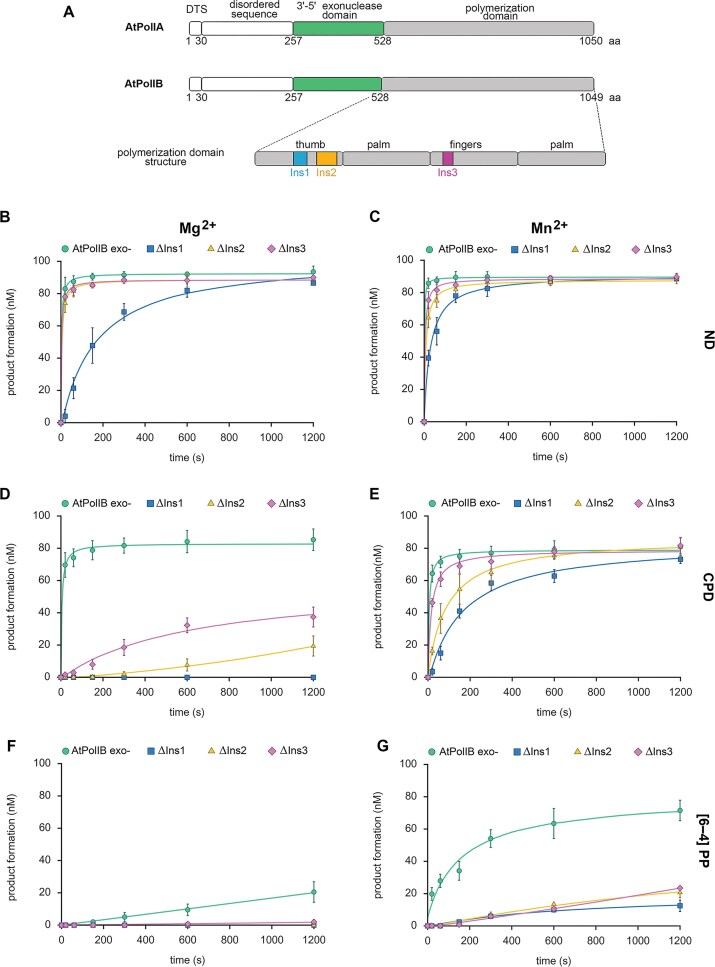
Polymerization activity on normal and damaged DNA of AtPolIB exo− and Insert mutants in the presence of Mg^2+^ or Mn^2+^. (**A**) Domain organization of AtPolIA and AtPolIB displaying a dual targeting sequence (DTS), a disordered sequence, a 3′–5′ exonuclease domain, and a polymerization domain where Ins1, Ins2, and Ins3 are depicted in cyan, orange, and pink, respectively. Primer extension assays on non-damaged (ND) templates in the presence of Mg^2+^ (**B**) or Mn^2+^ (**C**). ΔIns1 exhibits a reduced polymerization activity in the presence of Mg^2+^ compared to the rest of mutants or AtPolIB exo−. (**D**) Extension of CPD template indicating an inefficient TLS activity of ΔIns1, ΔIns2, and ΔIns3 in the presence of Mg^2+^, while Mn^2+^ restored a strong CPD extension in the different mutants (**E**). (**F**) Mg^2+^ was unable to promote TLS on [6–4] PP on the different deletion mutants and almost abolished on AtPolIB exo−. (**G**) Mn^2+^ enhances [6–4] PP TLS of AtPolIB exo− but this activity is still residual on the deletion mutants. Error bars represent the standard deviation from three repeats.

In previous works we reported that AtPolIs contain three insertions conserved among plant organellar DNAPs and demonstrated that they are involved in DNA repair and maintenance [23, 27, 29]. Here we aimed to evaluate the role of these insertions in AtPolIB to bypass CPD and [6–4] PP. For this, we constructed AtPolIB exo− individual deletion mutants (ΔIns1, ΔIns2, and ΔIns3) (Fig. 8A) and tested their DNA synthesis on the ND, CPD, and [6–4] PP templates using either Mg^2+^ or Mn^2+^ (Fig. 8). AtPolIB, ΔIns2, and ΔIns3 displayed comparable polymerization rates on the ND template, conversely; ΔIns1, presented a slower primer extension. Mn^2+^ drastically enhanced DNA synthesis of ΔIns1, AtPolIB exo− slightly improved the ability, while ΔIns2 and ΔIns3 remained almost similar compared to reactions incubated with Mg^2+^ (Fig. 8B and C).

The polymerization rate of AtPolIB exo− on CPD was similar with either Mg^2+^ or Mn^2+^. Deletion of Ins2 and Ins3 almost abolished the CPD TLS activity, as the product formation was very limited after long incubation times, while ΔIns1 disrupted the TLS when incubated with Mg^2+^ (Fig. 8D). Interestingly, Mn^2+^ restored CPD TLS ability to different extents on the three inserts (AtPolIB exo→ΔIns3>ΔIns2>ΔIns1), with ΔIns3 reaching a close polymerization rate as AtPolIB exo− (Fig. 8E). The presence of Mg^2+^ was not enough to stimulate [6–4] PP TLS of inserts/deletions and only an inefficient product formation is monitored by AtPolIB exo− after long incubation (Fig. 8F). As in CPD, Mn^2+^ also supported [6–4] PP extension by AtPolIB exo− but was unable to reestablish an efficient polymerization rate on the inserts deletions (Fig. 8G). These results suggest that the inserts are not essential for normal DNA synthesis, although deletion of Ins1 reduced AtPoll’s polymerization rate, they are necessary for synthesis across CPD. While in the presence of Mn^2+^, the insertions are only essential for extending [6–4] PP but not CPD.

During the primer extension assays, we noticed that ΔIns1 generated shorter products on ND template than the rest of deletions and AtPolIB exo− when incubated with Mg^2+^, ranging from 42 to 49 nt (Supplementary Fig. S9B). We further explored whether AtPolIB exo− and insert deletions are incorporating or skipping the CPD and [6–4] PP lesion. For this, we quantified extension products in reactions containing Mn^2+^. AtPolIB exo− and ΔIns3 are directly incorporating opposite UV lesions as we monitored the expected 49-nt extension product. While ΔIns2 would be conducting a moderate slippage page mechanism on [6–4] PP showing bands from 47 to 49 nts and no slippage on CPD. Interestingly, ΔIns1 generated extension bands ranging from 45 to 49 nt on CPD and non-damaged templates, indicating also a possible role in processivity (Supplementary Fig. S9C).

Overall, the products quantification indicates that ΔIns1 exhibits a strong slippage mechanism, while ΔIns2 moderately would be skipping the lesion, and AtPolIB exo− and ΔIns3 incorporate direct opposite of the CPD and [6–4] PP lesions.

### Insertions in the polymerization domain are involved in DNA binding

**Figure 9.**
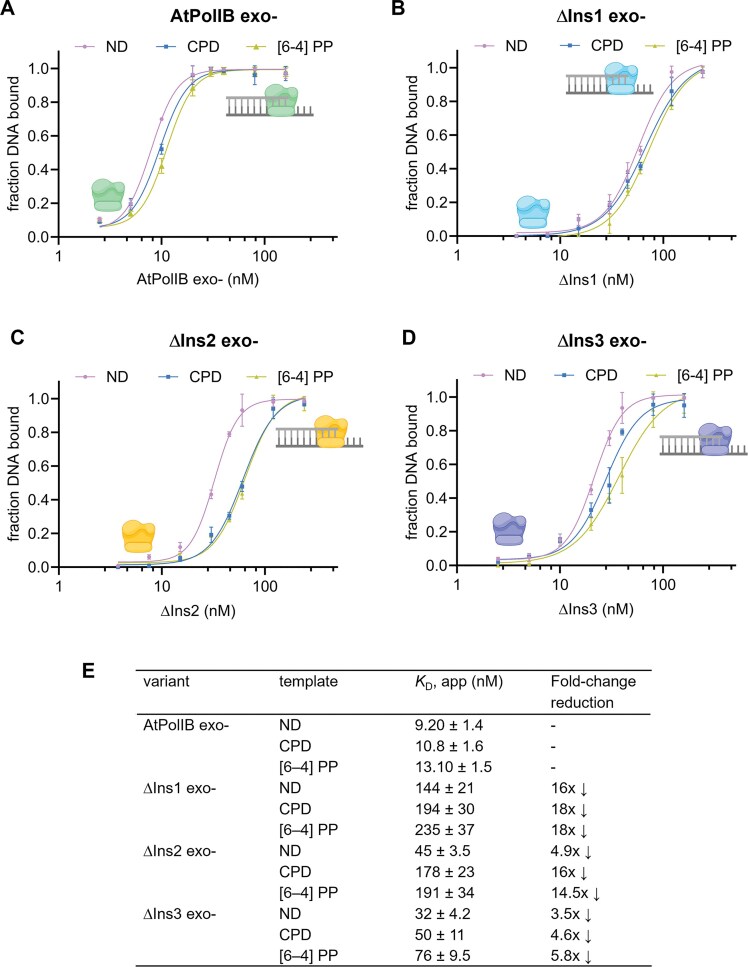
DNA-binding affinity of AtPolIB exo− and Inserts mutants on normal and damaged DNA. Quantification of EMSA data showing DNA binding on ND, CPD, and [6–4] PP by AtPolIB exo− (**A**), ΔIns1 (**B**), ΔIns2 (**C**), and ΔIns3 (**D**). (**E**) *K*_D_ values for normal and damaged DNA of each AtPolIB variant are derived from panels (A–D). DNA binding analysis was determined using data from Supplementary Fig. S10. Error bars represent the standard deviation from three repeats.

One important factor for lesion bypassing is the strong binding affinity to stabilize lesioned substrates in the polymerization site. Based on evidence that the insert mutants in AtPolIB are involved in UV-induced lesion extension, we evaluated whether they are playing a role in DNA-binding affinity using ND, CPD, and [6–4] PP templates annealed to the primer N (Supplementary Fig. S10). We found that AtPolIB exo− displayed a similar DNA binding affinity on the normal and damaged DNA substrates (9, 11, and 13 nM for ND, CPD, and [6–4] PP, respectively) (Fig. 9A and E). *K*_D_ values of AtPollB exo− on the non-damaged substrate (9 nM) contrast with those reported for Ggp5/thioredoxin, which range from 60 to 90 nM [37, 38], representing 6 to 10 times higher DNA binding affinity.

On normal DNA, ΔIns1 drastically reduced DNA binding by 16-fold compared to AtPolIB exo−, while a more detrimental affinity is observed for damaged templates (Fig. 9B and E). ΔIns2 showed similar reduction on CPD and [6–4] PP (16 and 14.5-fold) with a moderate five-fold decrease on the normal DNA (Fig. 9C and E). ΔIns3 was the least affected on DNA binding, with 3.5-, 4.6-, and 5.8-fold decrease for normal, CPD, and [6–4] PP substrates, respectively (Fig. 9D and E). Overall, these findings suggest that these inserts are participating in DNA binding, with ΔIns1 playing a more important role in DNA stabilization, which is consistent with our data where this mutant displays the lowest polymerization rate among the three inserts deletion.

## Discussion

UV-induced DNA lesions, such as CPDs and [6–4] photoproducts ([6–4] PPs), impose major hurdles for DNA replication. In nucleus, the bulking dimers are repaired by the nucleotide excision repair (NER) pathway. DNA replication through unrepaired lesions would switch DNA replicase with a group of specialized translesion synthesis (TLS) polymerases that can synthesize opposite to damaged template. Plant organellar DNA contains substantial amounts of UV-induced lesions but lacks NER and other comparable repair enzymes. Thus, the *Arabidopsis thaliana* organellar DNA replicases, AtPolIA and AtPolIB, inevitably encounter UV lesions during DNA synthesis.

In the nucleus, CPD bypass is carried out primarily by DNA polymerase η (Pol η). Pol η bypasses CPDs with high efficiency but low fidelity, with incorporation error rates opposite the 3′-T and 5′-T of CPDs of ~10% and 1%, respectively. Furthermore, synthesis processivity of Pol η across CPDs is higher only at the dimer and the flanking regions, including positions −1 and +1, but drops rapidly at +2. This suggests that Pol η is inefficient at extending primers beyond the lesion [39]. Here, we report that AtPolIA and AtPolIB can bypass CPDs with efficiencies comparable to those of Pol η and with similar error rates. However, unlike Pol η, AtPolIA and AtPolIB bypass CPDs with high processivity at the lesion and beyond, making them effective both as inserters and extenders across these lesions.

Contrarily, bypassing synthesis across [6–4] PP is more challenging: Pol η can only insert a single nucleotide opposite to 3′-T of 6–4 PP with <2% efficiency; Pol ι can insert one or two nucleotides opposite to the 3′-T and the 5′-T of [6–4] PP, then relies on Pol θ to perform low-efficiency extension, with an efficiency <5% [40]. Here we report that the AtPolls exhibited limited translesion synthesis across [6–4] PP with efficiency of ∼12%. However, silencing their 3′–5′ exonuclease activity increased their TLS activity to greater than 80%, reaching a level comparable to that on undamaged DNA templates.

The replicative T7 DNAP is unable to replicate on CPD or [6–4] PP lesions either in the presence of Mg^2+^ or Mn^2+^ while the exonuclease-deficient variant exhibits a very inefficient UV-induced lesion bypassing [41, 42]. *Escherichia coli* DNAP I displays a moderated bypassing ability on CPD but is blocked by [6–4] PP [43]. The mitochondrial replicative human Pol γ bypasses CPDs only in the presence of Mn^2+^ but it is unable to extend a [6–4] PP with either Mg^2+^ or Mn^2+^ even if the exonuclease activity is removed [36], indicating that exonuclease suppression is not the only factor to trigger UV-induced lesion TLS. These observations suggest a novel mechanism used by the replicative AtPolIs when a [6–4] PP or CPD is encountered, switching from a replicase to a translesion DNA synthesis DNAP.

**Figure 10.**
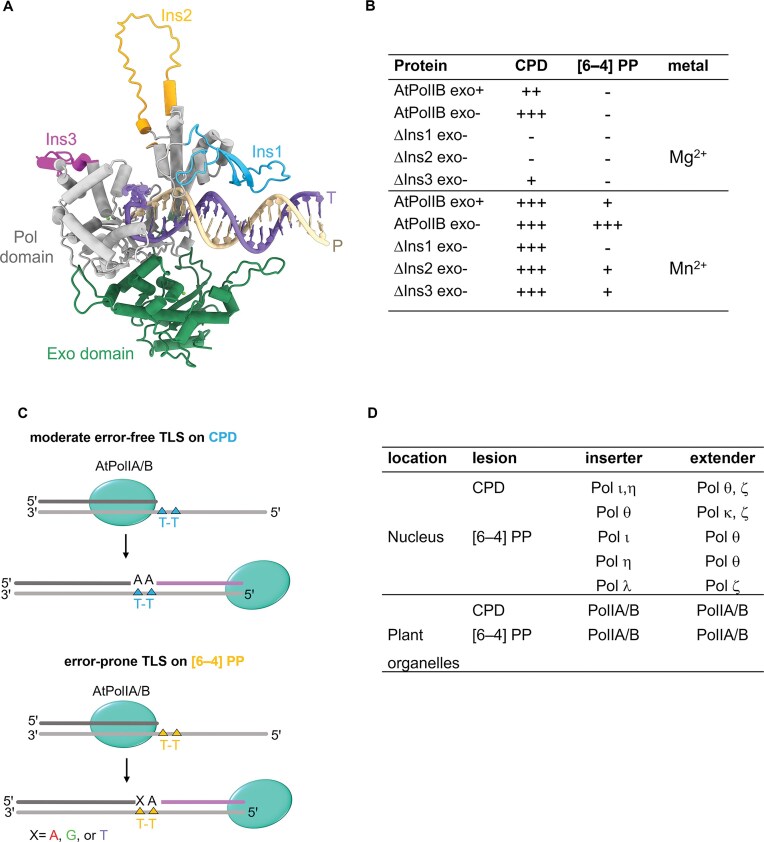
Proposed model of the Translesion DNA synthesis across UV lesions by plant organellar DNA polymerases. (**A**) AlphaFold prediction of AtPolIB (At3g20540) complexed with a primer/template DNA. Insertion 1 is located from residues 576 to 621 (blue), insertion 2 is mapped from residues 648 to 712 (orange), and insertion 3 is from 844 to 869 (magenta). (**B**) Table indicating the TLS activity for each AtPolIB-derived variant in the presence of Mg^2+^ or Mn^2+^. TLS was ranked as limited (+), moderated (++), and strong (+++) TLS activity. (**C**) Cartoon indicating the TLS pathway used by AtPolIs for CPD and [6–4] PP. (**D**) Table indicating the inserter and extender specialized DNA polymerases involved in CPD and [6–4] PP extension in nucleus compared to plant organelles where AtPolIs plays both roles during UV-induced DNA lesion.

In nucleus CPD and [6–4] PP bypass requires the coordinated action of two TLS DNA polymerases: the inserters that incorporate nucleotides opposite the lesion and the extenders that continue further lesion extension [40, 44–47]. Our results indicate that plant organellar DNA polymerases represent the first evidence of a replicative DNAP equipped with these abilities to overcome CPD with high efficiency and with potential to extend [6–4] PP using Mn^2+^ (Fig. 10D). Our results correlate with the fact that POPs are more related to TLS DNAPs such as Pol θ and Pol ν (Supplementary Fig. S11), which have evolved key structural regions in the polymerization domain to accommodate bulky DNA templates, and not with the metazoan replicative mitochondrial DNAP or with T-odd bacteriophages [40, 48, 49]. Combining our findings, we propose that AtPolls, and particularly AtPolIB, have evolved four features to achieve TLS on CPD and [6–4] PP lesions: (i) intrinsic promiscuous polymerase active site, (ii) the presence of modest exonuclease activity, (iii) a strong binding affinity for the primer-template, and (iv) use of divalent metal ions switching toward Mn^2+^ to modulate the polymerase active site and the three unique insertions found in POPs to accommodate UV-induced DNA lesions.

### AtPolIs presents more promiscuous polymerase active site than conventional replicases and novel inserts in the polymerization domain

In contrast to replicative DNA polymerases, an intrinsic feature of TLS polymerases is their low fidelity for nucleotide incorporation, lack of a 3′–5′ exonuclease domain, and a wide active site to accommodate bulky DNA lesions [50, 51]. Members of the family Y TLS DNA polymerases share a widened active site; for instance, in Pol η, the CPD is perfectly accommodated in the Pol site [52]. The assembly of a high-fidelity and tightly constrained active site during nucleotide incorporation in family A DNAPs [53, 54] is challenged by *Helicobacter pylori* DNAP I, *Entamoeba histolytica* DNAP I [55, 56], human Pol θ [57], and Pol ν [58], which bypass abasic sites and thymine glycol while executing nucleotide incorporation with low fidelity. Human Pol ν exhibits low fidelity due to specific amino acids of the α-helix O; however, those residues are not conserved in AtPolIs [49, 59]. In contrast to canonical DNAP I (Supplementary Fig. S11A), human Pol ν and Pol θ harbor three specific insertions in the polymerization domain, which enlarge the polymerase sites [25]. In Pol ν and Pol θ, Ins1 is mapped in the tip of the thumb and associated with DNA binding and processivity as it is in contact with DNA [59, 60] (Supplementary Fig. S11B and C). Ins2 is located at the base of the thumb and palm domains and in Pol θ, where it has been demonstrated to play a role in TLS activity [25] (Supplementary Fig. S12B). Ins3 is found at the backside of the palm domain and in Pol θ is prolonged toward the pseudoexonuclease domain, away from the DNA binding regions; however, deletion of this insert abolished lesion bypassing activity [25] (Supplementary Fig. S12C). Overall inserts in Pol θ are longer than the same inserts in Pol ν [52].

Plant organellar DNAPs seem to have evolved features found in replicative and TLS DNAPs. AtPolIs present contrasting fidelity for nucleotide incorporation, nearly 20-fold lower fidelity for nucleotide incorporation than their human counterpart, Pol γ [33]. Although both AtPolIs share ~75% amino acid identity, AtPolIB has been associated with DNA repair and maintenance mechanisms, consistent with its low fidelity during nucleotide incorporation compared to AtPolIA, which is proposed as the replicative DNAP in plant organelles [33, 61]. The unique ability of AtPolIs to bypass UV-induced DNA damages would be a result of novel structural modifications in the polymerization domain to accommodate bulky lesions. AtPolIs, like other POPs, also contain three unique insertions in the polymerization domain that resemble the inserts in human Pol θ and Pol ν but are absent in the canonical bacterial DNAP I (Supplementary Fig. S11). Despite these inserts playing similar roles in Pol θ and AtPolIs, they are not evolutionarily related. In AtPolIs, Ins1 is in the thumb domain, and it is the only one overlapping with Pol θ Ins1 [23] (Supplementary Fig. S11C and D). Our AlphaFold modeling shows that Ins1 is directly contacting the DNA (Supplementary Fig. S12A), which correlates with a decreased DNA binding ability, low polymerization rate, and abolition of the TLS activity in the Δ Ins1 variant (Figs 8 and 9). Ins2, with a highly flexible region, is the largest insert positioned in the middle of the thumb domain and connected with the helix H2 in the DNA vicinity (Supplementary Fig. S12B). Although this insert does not directly interact with DNA, a deletion resulted in a DNA-binding reduction and eliminated the TLS function (Figs 8 and 9). Ins3 is mapped in the finger domain and based on our AlphaFold analysis is distant from the DNA binding site but connected to the conserved O helix responsible for the template and incoming nucleotide accommodation (Supplementary Fig. S12C). Deletion of the Ins3 moderately lowered DNA-binding affinity and exhibited similar polymerization rate on normal DNA compared to AtPolIB exo−; however, the TLS ability was negatively altered (Figs 8 and 9). Δ Ins3 was the least affected of the inserts, consistent with its distal position from the pol site. Overall, when we deleted individual insertions, the TLS activity on both CPD and [6–4] PP disappeared, while replacing Mg^2+^ for Mn^2+^, only rescued the TLS on CPD, suggesting that these insertions may influence the active site dynamics, creating a more permissive active site or generating a clamp-like structure to encircle the DNA, allowing the extension of very distorting DNA damage. We also compared an AlphaFold model of AtPolIB to T7 DNAP structure in complex with a CPD-containing DNA. We found that the Q helix, which prolongs from the catalytic site toward the 5′-T of CPD, is longer in AtPolIB than in T7 DNAP (Supplementary Fig. S13). We speculate that in contrast to T7 DNAP, in AtPolIB this helix would be preventing the DNA lesion bending, ensuring the bulky lesion accommodation in the catalytic pocket. Further structural analyses will be required to shed light on how key elements such as inserts and Q-helix are positioned in a ternary complex with lesioned DNA.

### AtPolIs exhibit low exonucleolytic activity

3′–5′ exonuclease activity imposes a barrier to translesion DNA synthesis by excising inserted bases opposite DNA lesions and preventing further extension [62]. Studies comparing exonuclease-proficient and exonuclease-deficient members of the family A DNAPs show that removing the editing domain enables TLS on CPDs and other DNA lesions with different efficiencies [34, 42, 43]. Although AtPolls are replicative DNAPs in plant organelles, in previous studies we reported that their 3′–5′ exonuclease on dsDNA is lower than their counterpart in animals, Pol γ [33, 36, 63]. This editing activity on AtPolIs also contrasts with the potent 3′–5′ exonucleolytic activity of the T7 DNAP-thioredoxin complex that is calculated to be near 10 000-fold more active [64, 65]. In contrast to T7 DNAP, which uses structural motifs to guide the 3′-end of a primer into the exonuclease active site, AtPolIs lack those to direct primer shuttling from the polymerase into the exonuclease active site [66]. Thus, CPD lesion bypassing by AtPolls would rely on other elements, including the modest exonucleolytic activity supporting the idea that DNAPs with strong editing activity, such as T7 DNAP. can bypass CPD only when the exonuclease activity is deleted [42, 67]. Excision rates of AtPolIs on normal and damaged substrates demonstrate that AtPolIs contains a modest exonuclease activity and that AtPolIB displays about two-fold lower activity than AtPolIA, which supports the hypothesis that one is the true replicative DNAP while the other is more involved in DNA maintenance. Overall, both AtPolIs increase the excision rate when primer is placed opposite the UV lesions or a couple bases past, but interestingly, the excision ability is comparable to that observed for normal DNA when the primer is right before the lesion or at the +3 position after (Table 2 and Supplementary Fig. S2). These findings indicate that AtPolIs can detect intermediate incorporation products, which would preferentially digest down to one position before the lesion where detection becomes weaker as replication progresses beyond the lesion.

Both AtPolIs preferentially added two adenines opposite CPD and continued an efficient extension (Supplementary Fig. S7B and C). Contrarily, during [6–4] PP synthesis, both AtPolIs added A, G, or T opposite the 3′-T of the lesion, becoming a barrier for the next incorporation, which results in the rate-limiting step due to formation of a T-G pair (Fig. 7 and Supplementary Fig. S7F–I *right*). Although AtPolIs reduce the nucleotide incorporation on the 5′-T of [6–4] PP, when a dAMP is inserted at the 3′-T of the lesion, a second dAMP is incorporated at the 5′-T of [6–4] PP. Interestingly, when a dTMP is misincorporated at the 3′-T of the lesion, AtPolIs places a dCMP at the 5′-T of [6–4] PP, creating a second mismatch (Supplementary Fig. S8). We also observed mismatch formation in N + 2 and N + 3 primers, in which three and two dTMPs are incorporated, resulting in a T-G mismatch at the +2 position after the [6–4] PP (Supplementary Fig. S7 F–I). After quantifying the expected full-length product, we inclined more for a translocation barrier mechanism, resulting in a misincorporation event rather than slippage, as we observed correct matches before the T-G mismatch. We hypothesize that AtPolIs tend to generate mismatches by incorporating less voluminous bases to reduce the DNA distortion caused by the lesion. This idea is supported by the results where two consecutive misincorporations are observed opposite the [6–4] PP created by purine bases.

These observations explain why AtPolIs extend a [6–4] PP with very limited efficiency, likely due to a shuttling of these mismatches and nonproductive intermediates originating at the 3′-T of the lesion (Fig. 7C) toward the exonuclease domain, reducing the probability of further extensions and corroborating results using exonuclease-deficient variants exhibiting stronger TLS activity.

### AtPolIs have a strong binding affinity for the primer-template

Enhanced DNA binding is a key feature for an efficient translesion DNA synthesis activity, coming from the DNA polymerase itself or the interaction with protein partners. Numerous studies have shown that the processivity factor PCNA increases the ability of replicative and TLS DNA polymerases to bypass DNA lesions [68–71]. DNA binding analysis of AtPolIB shows a high DNA binding affinity with similar *K*_D_ values for normal and UV-lesion-containing DNA substrates, and although deletion of each of the three inserts in the polymerization domain reduced the DNA affinity, ΔIns1 displays the largest decrease on both lesioned and normal DNA (Fig. 9), which correlates with the AlphaFold models predicting its direct DNA interaction (Supplementary Fig. S12). Based on this evidence, we propose that enhanced DNA binding activity—an important factor to stabilize voluminous DNA damage in the polymerization site—is conferred by the novel inserts in AtPolIs. Our results strongly suggest that AtPolIs TLS activity resides on the three insertions in the polymerization domain (Fig. 10A and B). This feature resembles that found in human Pol θ, where its insertions are implicated in primer-template stabilization during TLS activity on abasic sites and thymine glycols [72]. Mutants of AtPolIB lacking the individual inserts recovered the CPD TLS activity only in the presence of Mn^2+^, which would suggest that the remaining inserts undergo structural reorganizations expected due to their highly flexible region according to AlphaFold predictions and are potentially crucial for DNA encircling to increase DNA binding affinity.

Nonetheless, AtPolIs exo+ bypasses [6–4] PP with low efficiency and may require protein interactor to strengthen the TLS activity. The replicative T7 DNAP is unable to bypass CPD by itself, while forming a complex with the bacteriophage T7 helicase enables the bypassing activity [73]. TLS by this complex suggests that the intrinsic T7 DNAP TLS ability would be assisted by a protein that increases its DNA binding affinity to keep the lesioned DNA in the pol site while reducing shuttling to the exonuclease site, allowing extra incorporation attempts.

We hypothesize that AtPolIs TLS activity would be stimulated in a similar fashion by their immediate cognate, the primase-helicase AtTwinkle. In previous reports it has been demonstrated that AtTwinkle specifically interacts with AtPolls to form a T7-like replisome complex in which AtPolIs efficiently extend newly synthesized primers by AtTwinkle [74, 75]. Suggesting that this complex would be also formed to replicate UV DNA lesions. Similar to T7 replisome, AtTwinkle would suppress the already limited AtPolIs exonuclease activity by stabilizing the post-translocation state and improving DNA binding affinity for [6–4] PP lesion, allowing several incorporation attempts to increase [6–4] PP extension.

### Manganese ions modulate the flexibility of the polymerase active site

Mn^2+^ can activate activities of many DNA polymerases at lower concentrations than Mg^2+^ as it binds tighter to the protein active sites while modifying DNA synthesis fidelity [76–79]. However, Mn^2+^ can also enhance translesion DNA synthesis of other specialized DNA polymerases. Mn^2+^ improves the efficiency of lesion bypassing across diverse DNA roadblocks, enabling changes in DNA substrate specificity, coordination geometries and reducing energetic barriers [80–83]. A recent study demonstrated that Mn^2+^ can stimulate CPD TLS activity of the replicative human Pol γ but fails to promote [6–4] PP extension on the wild type or exonuclease-deficient variant [36]. Interestingly, we found that both wild-type AtPolIs displayed a strong TLS on CPD in the sole presence of Mg^2+^ or Mn^2+^. While Mn^2+^ triggers a limited ability to bypass [6–4] PP damage and becomes robust when exonuclease activity is removed. To our best knowledge, there is not report of other exonuclease-deficient replicase or TLS DNAP able to incorporate and extend [6–4] PP with high efficiency.

Mg^2+^ and Mn^2+^ are essential micronutrients necessary for plant growth. The concentrations of free Mg^2+^ in chloroplasts range from 0.5 to 2.0 mM [84] and it can increase in a light-dependent manner up to 5 mM [85–87], consistent with our results where the increment of Mg^2+^ facilitated the TLS activity on CPD substrates (Fig. 2). Whereas Mn^2+^ concentrations in plants rank between 0.1 and 1.5 mM, under stress conditions they can reach up to 2.0–2.5 mM [84, 87, 88]. Plant regulates free-Mn^2+^ availability to reduce potential ROS damages, for which most of the Mn^2+^ reserves are found in vacuoles [89, 90]. It has been observed that increased manganese concentration contributes to UV-induced damage tolerance by activating enzymes involved in ROS removal mitigation [91]. We hypothesize that this abnormal cellular condition can favor other enzymes such as AtPolIs, which, according to our results, can select Mn^2+^ over Mg^2+^ from a mixture to enable TLS activity on [6–4] PP (Supplementary Fig. S4). AtPolIs performs a moderate error-free TLS on CPD, while on [6–4] PP becomes mutagenic. Reduced fidelity is trade-off for a better outcome for the organism than a fork replication collapse. Lesion bypassing mutation-derived would be subsequently repaired, as it has been demonstrated that UV-induced DNA damage in plant organelles remains low, indicating that an unknown UV lesion repair mechanism is present [16]. Individual deletion of the three inserts in AtPolIs is unable to efficiently extend both CPD and [6–4] PP lesions in the presence of Mg^2+^, while the presence of Mn^2+^ restores the TLS only on CPD (Fig. 8). One conclusion is that Mn^2+^ may play a key role in reshaping these critical regions, allowing a more permissive catalytic pocket by changing coordination geometries to accommodate bulky lesions, reduced dNTP discrimination, which would ensure lesion bypassing.

Overall, we conclude that AtPolIs have evolved features from TLS DNA polymerases while maintaining characteristics from replicases. This evolution was a result of replicating genomes in chloroplasts and mitochondria, environments with high DNA-damaging agents such as ROS and UV light. One of the key features is the three inserts in the polymerization domain, which enable strong DNA binding affinity and bypassing activity. Another feature is the more promiscuous polymerase site, which allows potential bulky lesion accommodation and modest exonuclease activity compared to other replicative DNA polymerases. And finally, the ability to use Mn^2+^ to enhance different activities and key protein region reshaping, all this to ensure an efficient TLS activity.

## Supplementary Material

gkag707_Supplemental_File

## Data Availability

The data underlying this article are available in the article and in its online supplementary material.
